# Entropic Dynamics of Mutations in SARS-CoV-2 Genomic Sequences

**DOI:** 10.3390/e26020163

**Published:** 2024-02-14

**Authors:** Marco Favretti

**Affiliations:** Dipartimento di Matematica “Tullio Levi-Civita”, Università degli Studi di Padova, 35123 Padova, Italy; favretti@math.unipd.it

**Keywords:** single nucleotide variations, C–T bias, Markov models, mean field dynamics, master equation, stochastic thermodynamics

## Abstract

In this paper, we investigate a certain class of mutations in genomic sequences by studying the evolution of the entropy and relative entropy associated with the base frequencies of a given genomic sequence. Even if the method is, in principle, applicable to every sequence which varies randomly, the case of SARS-CoV-2 RNA genome is particularly interesting to analyze, due to the richness of the available sequence database containing more than a million sequences. Our model is able to track known features of the mutation dynamics like the Cytosine–Thymine bias, but also to reveal new features of the virus mutation dynamics. We show that these new findings can be studied using an approach that combines the mean field approximation of a Markov dynamics within a stochastic thermodynamics framework.

## 1. Introduction

The sudden outburst in 2019 of the COVID-19 pandemic has generated a prompt and powerful reaction in the scientific and political community to fight against the worldwide menace represented by the virus ([1]). One of the first actions undertaken was the deployment of a large genome sequencing effort, which has generated a very large database (about $10^{6}$ sequence as of September 2023) of SARS-CoV-2 sequences in a short timespan. This unprecedented data richness, along with the certain identification of the ancestral virus sequence, has allowed scientists to undertake a detailed scrutiny of the virus evolution in the human host population. The main effort in the genetic research has been axed on functional domain analysis to identify regions in the sequence which are related to protein formation and thus responsible of key virus characteristics such as spreading speed or sensitivity to vaccine or drug treatments.

In this paper, we take a different approach, which can offer a complementary viewpoint on the dynamic of the virus mutation mechanism. In this study, we use the National Center for Biotechnology Information (NCBI, www.ncbi.nlm.nih.gov) database. We downloaded all of the complete RNA sequences with no unknown characters and with the same length (29,903 characters) of the Wuhan reference sequence classified as NC045512.2 in NCBI database. There were about 5600 sequences meeting the above criteria at the retrieval date of February 2023. These constitute the dataset for this study. Moreover, we reduce the high complexity of the nearly 30,000-base-long genomic viral sequences to the study of the four-dimensional probability vector $p=(p_{A},p_{C},p_{G},p_{T})$ of the A,C,G,T base frequencies.

In the following, we speak of entropy of a sequence, intending the Shannon entropy of the associated base frequency vector (*p* or *q*) for the sake of simplicity. We can then compute the entropy of the most ancient known virus sequence (the one found in Wuhan, China), which we denote with h(q); the entropy h(p) of (any of) the mutated sequences; and the relative entropy D(p|q) between the reference sequence and a mutated one. See, e.g., [2] for a gentle introduction to these notions of Information Theory.

The choice of the Shannon entropy as a statistical indicator of a sequence implies that two sequences that differ in a simple permutation in the bases are indistinguishable; moreover, their relative entropy is zero. Therefore, with the approach chosen in this study, only an accumulation of mutations that changes the base frequency is appreciable. We are aware that this is a drastic simplification of the actual mutation mechanism; nevertheless, we find that such a simple model of mutations is capable to reveal new and unexpected features of the mutation dynamics. Here is the plan of the paper.

In Section 2, we show that the accumulation of mutations in the sequence decreases the entropy of the sequence with respect to the ancestral one. This means that the mutations necessarily further increase the original unbalance of the proportions between the bases of the reference sequence ($q_{C}<q_{G}<q_{A}<q_{T}$), enhancing the unbalance $p_{T}>p_{C}$ and $p_{A}>p_{G}$, a phenomenon already reported in the literature (see, e.g., [3,4,5]), the so-called C→T bias. We find that the decrease in entropy has a analytically computable lower bound, called the minimal entropy curve, which is tight for many of the sequences in the dataset.

In Section 3, we investigate the dynamic of mutations introducing a simple Markovian model, which is used in population dynamic studies and which is akin to the classical Eherenfest urn model in statistical thermodynamics. We compute the mean field approximation of the Markovian dynamics, which gives a master equation type equation. We then compare the evolution of the entropy along the mean field solution with the minimal entropy curve. Note that, unlike the theoretical minimum entropy curve, which is based only on the knowledge of *q*, the Markovian model of mutation dynamic requires the knowledge of the Markov matrix *P* of transitions and trasversions, which is computed from the dataset in Section 5. This additional piece of information allows us to track the entropy evolution more closely.

In Section 4, we look at our Markovian dynamic model using the stochastic thermodynamics framework. This allows to describe the mutation bias—which acts as a drift term in the evolution of the base frequency—as the effect of the interaction of a small thermodynamic system with a thermal bath. We can, thus, compute the entropy flow and entropy production term related to the stochastic evolution (see [6,7,8]). Even if we can think of our set of sequences as a thermodynamic system only by analogy, this identification is useful to quantitatively describe the system’s entropy evolution.

## 2. Computation of Minimal Entropy Curve

**Figure 1:**
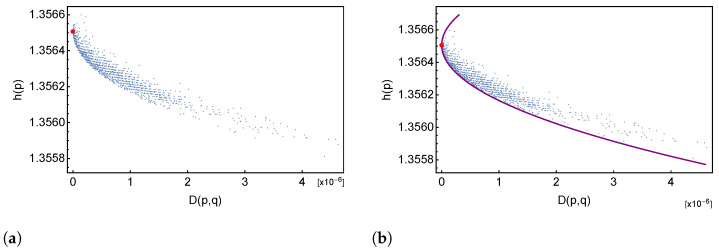
(**a**) Plot of the entropy vs. relative entropy of the sequences in the dataset (blue dots); red dots represent the entropy of the reference (Wuhan) sequence. (**b**) same as in (a); purple curve represents the minimum entropy curve.

We consider the base frequency *p* of a sequence as a random variable, because the initial RNA sequence is subject to the error-prone copying mechanism. We can then ask ourselves if h(p) increases or decreases with time, or if it fluctuates around its initial value h(q). Since mutations accumulate with time, it is natural to use the relative entropy D(p|q) as a “time variable” and investigate how the entropy h(p) changes with D(p|q). In Figure 1a, we plotted the entropy h(q) of the reference sequence *q* (red dot) and the entropy h(p) of the mutated sequences in the dataset as a function of their relative entropy “distance” D(p|q). A clear pattern emerges: the entropy is decreasing with the relative entropy. This is a clear indication that the mutations are non-random, otherwise the mutations would more likely affect the most abundant base and the resulting base frequency vector would be more “uniform”, hence with higher entropy. If the entropy is to decrease, this means that the effect of mutations has to further unbalance the initial base frequency vector *q*. In the sequel, we will address quantitatively this aspect of the mutation dynamic mechanism.

To start with, we want to determine if the decrease in entropy has a computable lower bound. This amounts to determine the probability *p*, which has minimal entropy h(p) over the set of probability distributions that satisfy the constraint D(p|q)=d and the normalization constraint. To this, we use the Lagrange multipliers method [9] for the Lagrange function (here, the index i∈E={A,C,G,T})

(1) $$G\left(p,\lambda ,\mu \right)=h\left(p\right)-\lambda \left(D\left(p|q\right)-d\right)-\mu \left(\sum_{i}p_{i}-1\right)$$

The necessary first order condition for the extremality $\partial G/\partial p_{i}=0$ for all *i* gives $p_{i}=C\left(\mu \right)q_{i}^{\frac{\lambda }{\lambda +1}}.$ By setting β=λ/(λ+1) and imposing the normalization constraint, we find the solution

(2) $$p_{i}\left(\beta \right)=\frac{q_{i}^{\beta }}{Z(\beta )}=\frac{q_{i}^{\beta }}{\sum_{i}q_{i}^{\beta }}$$

Note that for β=1 we have $p_{i}\left(1\right)=q_{i}$. The value of the multiplier β=β(d) is determined by the constraint D(p(β)|q)=d, which translates into the following equation:

(3) $$D\left(p\left(\beta \right)|q\right)=-h\left(p\left(\beta \right)\right)-\sum_{i}p_{i}\left(\beta \right)\ln q_{i}=\left(\beta -1\right)\sum_{i}p_{i}\left(\beta \right)\ln q_{i}-\ln Z\left(\beta \right):=f\left(\beta \right)=d$$

The function *f* has a minimum in β=1 with f(1)=0; so, for d>0, the equation f(β)=d has two solutions with $\beta_{1}\left(d\right)<1<\beta_{2}\left(d\right)$.

To ascertain if they provide a local constrained minimum or maximum for *h*, we invoke the second order sufficient conditions (see again [9]) on the hessian matrix of *G*: $\hat{p}$ is a local minimum (resp. maximum) if and only if $H_{p}G\left(\hat{p}\right)$ is positive (resp. negative) definite. In our case (here, $\delta_{ij}$ is the Kronecker symbol),

(4) $${\left(H_{p}G\right)}_{ij}=\frac{\partial^{2}G\left(p\right)}{\partial p_{i}\partial p_{j}}=\left(\frac{1}{\beta -1}\right)\frac{\delta_{ij}}{p_{i}}$$

hence the $\beta_{2}\left(d\right)>1$ solution of the equation f(β)=d yields a minimum, while the other $\beta_{1}\left(d\right)<1$ a maximum. If we plot the value of the entropy h(p(β(d)) along the two solutions $\beta_{2}\left(d\right)$ and $\beta_{1}\left(d\right)$ giving, respectively, the minimum and maximum possible value of the entropy h(p) for a given value of D(p|q)=d, we obtain the two branches of the violet curve in Figure 1b (upper branch has been cropped in the figure). We see that the lower bound is tight in the first part of the descent, and that there are mutated sequences that have minimal entropy.

**Figure 2:**
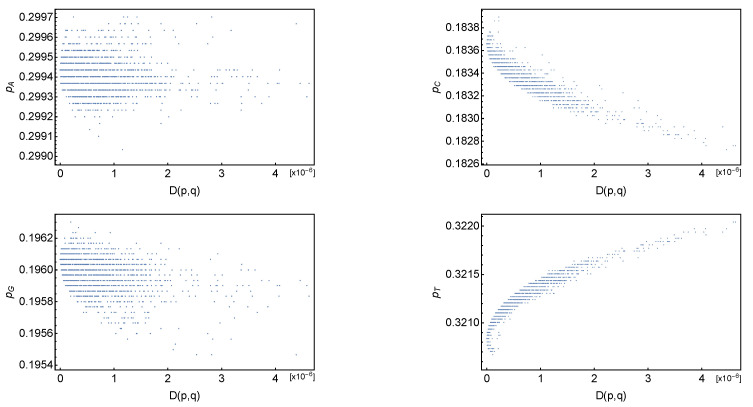
Left to right and top to bottom: plot of $p_{A}$, $p_{C}$, $p_{G}$ and $p_{T}$ base frequencies as a function of relative entropy distance from *q*.

In Figure 2, we computed the evolution of the base frequencies $\left(p_{A},p_{C},p_{G},p_{T}\right)$ with D(p,q). We see that there is a strong mutation bias favoring the substitution of C→T bases and perhaps a weak mutation bias G→A. A detailed study of the molecular nature of the bias is beyond the scope of this study; however, we notice that the above Formula (2) allows us to compute the evolution of the $p_{i}/p_{j}$ ratio for sequences that are close to the minimum entropy curve. Assuming that their base frequency vector is well described by (2), one has

(5) $$\frac{p_{i}}{p_{j}}=\frac{q_{i}^{\beta }/Z}{q_{j}^{\beta }/Z}={\left(\frac{q_{i}}{q_{j}}\right)}^{\beta (d)}$$

since β(d)>1, the initial unbalance $q_{i}/q_{j}$ increases with *d*. While it is understandable that the C–T mutation bias lowers the entropy of the mutate sequence, Figure 1b shows that the mutation dynamics drives the decrease of the entropy to the *minimum* possible value. To our knowledge, this is a new result.

From Figure 1b one sees that the minimum entropy curve represent a lower bound for the sequence’ entropy, which is saturated in the first part of the curve and which is loosened in the second part, giving evidence that there is some additional underlying mechanism in the mutation dynamics. In the following Section 3, we present a simple stochastic model to study this feature of the mutation dynamics.

## 3. A Stochastic Model of Mutation Dynamics

This kind of model is used in the Ehrenfest model of equilibrium thermodynamics (see, e.g., [10]) and in population dynamics [11] (see also [12] for the use of Markov models in mutation dynamics). We consider four urns (named A, B, C, D), each containing $n_{i}$ identical point particles with i∈E and $\sum_{i}n_{i}=N$. At each time step Δt, only one particle is randomly chosen from one urn and placed in one of the four urns. So, the change in the number of particles in urn *i* at time *t* is

(6) $$\Delta n_{i}\left(t\right)=n_{i}\left(t+\Delta t\right)-n_{i}\left(t\right)$$

with $\Delta n_{i}\left(t\right)\in \left\{-1,0,1\right\}$. Let $p_{i}=n_{i}/N$ be the probability that the chosen particle belongs to urn *i*, and let

(7) $$P_{ij}=Prob\left(i\to j|i\right),\;\mathrm{with}\;\sum_{j}P_{ij}=1$$

be the conditional probability that the particle in urn *i* at time *t* is moved to urn *j* at time t+Δt. Then, the average value of $\Delta n_{i}\left(t\right)$ is

(8) $$\langle \Delta n_{i}\left(t\right)\rangle =\sum_{j}p_{j}P_{ji}-p_{i}P_{ij}=\sum_{j}p_{j}P_{ji}-p_{i}={\left(\left(P^{T}-I\right)p\right)}_{i}$$

where $P^{T}$ is the transpose of *P* and I is the identity matrix. If the matrix *P* is independent of time, this model is a (time-homogeneous) discrete time Markov chain that can be used to describe the random variations in the base frequencies of the sequences. The following heuristic argument can be made rigorous (see, e.g., [13] and Appendix A). If the number of particles of the sequence *N* is sufficiently large, we can assume that the variance of the random variable $n_{i}$ is vanishing with *N*, so that

(9) $$\sigma_{i}=\langle {\left(n_{i}-\langle n_{i}\rangle \right)}^{2}\rangle \to 0\;for\;N\to \infty$$

Hence, $n_{i}\approx \langle n_{i}\rangle$ for large *N*. So, by multiplying (6) by 1/N

(10) $$\frac{1}{N}\Delta n_{i}\left(t\right)=\frac{1}{N}\left(n_{i}\left(t+\Delta t\right)-n_{i}\left(t\right)\right)$$

and taking the average, we obtain

(11) $$\frac{1}{N}\langle \Delta n_{i}\left(t\right)\rangle =\langle \frac{1}{N}\left(n_{i}\left(t+\Delta t\right)-n_{i}\left(t\right)\right)\rangle \approx p_{i}\left(t+\Delta t\right)-p_{i}\left(t\right).$$

If we take Δt=1/N as a time step (this means that a time *T*∼1 is the time required to move all the particle of the system on average), then we can write (11) as

(12) $$\frac{p_{i}\left(t+\Delta t\right)-p_{i}\left(t\right)}{\Delta t}\approx \frac{1}{\Delta tN}\langle \Delta n_{i}\left(t\right)\rangle =\langle \Delta n_{i}\left(t\right)\rangle .$$

In the limit N→∞, (12) becomes an equality and using (8), we obtain the following ODE for the probability *p*:

(13) $${\dot{p}}_{i}=\langle \Delta n_{i}\left(t\right)\rangle ={\left(\left(P^{T}-I\right)p\right)}_{i},\;i\in E.$$

Note that a probability distribution $p=(p_{A},\ldots ,p_{T})$ is stationary if $P^{T}p=p$. In the following, we set $W=P^{T}-I,\;\sum_{i}W_{ij}=0$, and we consider the Cauchy problem

(14) $$\dot{p}=Wp,\;p\left(0\right)=q.$$

The above equation is called mean field approximation of the Markov chain [13]. In statistical thermodynamics, it is known as a master equation-type dynamic [14]. Equilibria Wp=0 of the master equation coincides with above-introduced stationary distributions $P^{T}p=p$. One can easily show that the above Equation (14) can be rewritten using the matrix *W* as

(15) $${\dot{p}}_{i}=\sum_{j}W_{ij}p_{j}-W_{ji}p_{i}=\sum_{i}J_{ij}$$

where the quantity $J_{ij}=W_{ij}p_{j}-W_{ji}p_{i}$ is called probability current or thermodynamic flux term.

A simple check on (15) shows that if the matrix *W* is symmetric ($W_{ij}=W_{ji}$), then the uniform distribution is an equilibrium distribution, and if the matrix *W* is non degenerate, then it is the only equilibrium; therefore, the entropy tends to its absolute maximum value when the system approach the equilibrium. Therefore, if the system entropy is to decrease as in our case, the matrix *W* (hence *P*) has to be non-symmetric.

**Figure 3:**
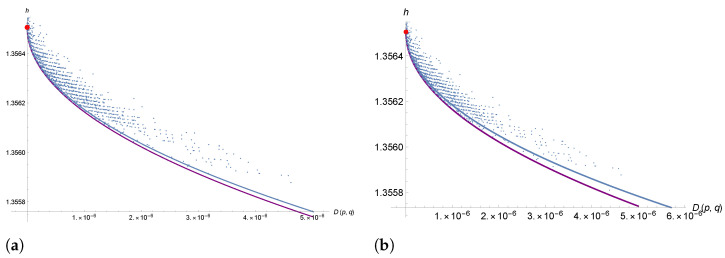
(**a**) Purple line: minimum entropy curve, blue line: entropy computed along the mean field solution for W=W(1). The mean field solution gives a better lower bound for the system entropy in the lower part of the curve. (**b**) The same as in (**a**) for W(40).

If *N* is large, we can assume that the mean field dynamics is a good approximation of the mutation dynamic mechanism. For our sequences, *N*∼3 × 10^4^, which gives a pretty good approximation. We can, therefore, compute h(p(t)) along a solution p(t) of the Cauchy problem (14), and compare its evolution with the plot of Figure 1b; see Figure 3 below. Note that, unlike the theoretical minimum entropy curve, which requires only the knowledge of *q*, the mean field model of mutation dynamic requires the knowledge of the Markov matrix *P*. In Section 5, we show how to compute *P* from the sequences of the dataset. Prior to this, in Section 4, we investigate this Markovian mutation dynamic model using a stochastic thermodynamics framework.

## 4. Stochastic Thermodynamic Interpretation of Entropy Decrease

Stochastic thermodynamics is a recent research field at the intersection of classical statistical thermodynamics with information geometry (see, e.g., [6,7]). Some new and old thermodynamic inequalities have been introduced and interpreted in terms of information geometry [15], and then applied to the description of “small” thermodynamic systems in the non stationary regime, like molecular motors. Stochastic thermodynamics seems thus to be a promising tool to study the RNA chain of nucleic acid mutation mechanism. We consider a probabilistic system, which has four states (A, C, G, T), and we suppose that the sequence base frequency $p=(p_{A},p_{C},p_{G},p_{T})$ evolves randomly due to its internal dynamics and due to the interaction with an environment, which is responsible of the bias or drift. We want to compute the time evolution of the entropy h(p(t)) along a solution of the Cauchy problem (14). We thus have

(16) $$\begin{array}{ccc} \dot{S}=\frac{dh}{dt} & = & \frac{d}{dt}\left(-\sum_{i}p_{i}\ln p_{i}\right)=-\sum_{i}{\dot{p}}_{i}\ln p_{i}=-\sum_{i,j}W_{ij}p_{j}\ln p_{i}=-\sum_{i,j}W_{ij}p_{j}\ln\frac{p_{i}}{p_{j}}\\ & = & -\frac{1}{2}\sum_{i,j}(W_{ij}p_{j}\ln\frac{p_{i}}{p_{j}}+W_{ji}p_{i}\ln\frac{p_{j}}{p_{i}})=\frac{1}{2}\sum_{i,j}\left(W_{ij}p_{j}-W_{ji}p_{i}\right)\ln\frac{p_{j}}{p_{i}}\\ & = & \frac{1}{2}\sum_{i,j}J_{ij}\ln\frac{p_{j}}{p_{i}} \end{array}$$

Now, write

(17) $$\ln\frac{p_{j}}{p_{i}}=\ln\frac{p_{j}W_{ij}W_{ji}}{p_{i}W_{ij}W_{ji}}=\ln\frac{p_{j}W_{ij}}{p_{i}W_{ji}}+\ln\frac{W_{ji}}{W_{ij}}=X_{ij}+\ln\frac{W_{ji}}{W_{ij}}$$

where $X_{ij}=\ln p_{j}W_{ij}-\ln p_{i}W_{ji}$ is the thermodynamic force. We can thus rewrite

(18) $$\dot{S}=\frac{dh}{dt}=\frac{1}{2}\sum_{i,j}J_{ij}X_{ij}+\frac{1}{2}\sum_{i,j}J_{ij}\ln\frac{W_{ji}}{W_{ij}}={\dot{S}}_{i}+{\dot{S}}_{e}.$$

The non-negative quantity

(19) $${\dot{S}}_{i}=\frac{1}{2}\sum_{i,j}J_{ij}X_{ij}=\frac{1}{2}\sum_{i,j}W_{ij}p_{j}\ln\frac{W_{ij}p_{j}}{W_{ji}p_{i}}\geq 0$$

is interpreted as the system’ entropy production therm. Note that $J_{ij}\left(p\right)=0$ for all i,j if *p* is the stationary distribution; therefore, the entropy production term vanishes when the system approaches the equilibrium distribution. The term with no definite sign

(20) $${\dot{S}}_{e}=\frac{1}{2}\sum_{ij}J_{ij}\ln\frac{W_{ji}}{W_{ij}}$$

is the entropy exchange (entropy flow) with the environment (heath bath). From Figure 1 we see that for our system we have $\dot{S}<0$ which from (18) necessarily implies that ${\dot{S}}_{e}<0$. Within this stochastic thermodynamic interpretation, the mutation bias act like a cold environment which lowers the entropy of the system. However, the necessarily non negative contribution ${\dot{S}}_{i}$ could induce a decrease of the entropy, which is *slower* that the one prescribed by the absolute minimum entropy curve of Figure 1b. In fact, the difference between the two curves in Figure 3 below is due to the positive contribution of the system entropy production term ${\dot{S}}_{i}>0$.

### 4.1. Log-Sum Inequality and the Entropy Production Term

To make this work self-contained, we briefly recall here a derivation contained in [7], which may be relevant for the interpretation of the entropy production term ${\dot{S}}_{i}$. Suppose that the master equation matrix $\hat{W}=\sum_{k=1}^{m}W\left(k\right)$ is the sum of *m* various contribution terms W(k), which describe the interaction of the system with different environments. Then, one can repeat verbatim the derivation in (16) for $\hat{W}$ and, subsequently, substitute definition (17) with the following one:

$$\ln\frac{p_{j}}{p_{i}}=\ln\frac{p_{j}\prod_{k=1}^{m}W_{ij}\left(k\right)W_{ji}\left(k\right)}{p_{i}\prod_{k=1}^{m}W_{ij}\left(k\right)W_{ji}\left(k\right)}=\sum_{k=1}^{m}\ln\frac{p_{j}W_{ij}\left(k\right)}{p_{i}W_{ji}\left(k\right)}+\ln\frac{W_{ji}\left(k\right)}{W_{ij}\left(k\right)}$$

Hence, (18) becomes

$$\dot{S}=\frac{1}{2}\sum_{ijk}J_{ij}\left(k\right)X_{ij}\left(k\right)+\frac{1}{2}\sum_{ijk}J_{ij}\left(k\right)\ln\frac{W_{ji}\left(k\right)}{W_{ij}\left(k\right)}={\dot{S}}_{i}+{\dot{S}}_{e}.$$

It is straightforward to rewrite the entropy production term ${\dot{S}}_{i}$ as

(21) $${\dot{S}}_{i}=\frac{1}{2}\sum_{ijk}J_{ij}\left(k\right)X_{ij}\left(k\right)=\frac{1}{2}\sum_{ij}\left(\sum_{k=1}^{m}W_{ij}\left(k\right)p_{j}\ln\frac{W_{ij}\left(k\right)p_{j}}{W_{ji}\left(k\right)p_{i}}\right)$$

Now, apply the log-sum inequality ([2], Cap.2)

$$\sum_{k=1}^{m}a_{k}\ln\frac{a_{k}}{b_{k}}\geq \left(\sum_{k=1}^{m}a_{k}\right)\ln\frac{\sum_{k}a_{k}}{\sum_{k}b_{k}}$$

which is valid for non-negative numbers $a_{1},\ldots a_{m}$, and $b_{1},\ldots ,b_{m}$. Then, (21) satisfies the inequality

(22) $${\dot{S}}_{i}=\sum_{k=1}^{m}{\dot{S}}_{i}\left(k\right)=\sum_{k=1}^{m}\frac{1}{2}\sum_{ij}\left(W_{ij}\left(k\right)p_{j}\ln\frac{W_{ij}\left(k\right)p_{j}}{W_{ji}\left(k\right)p_{i}}\right)\geq \frac{1}{2}\sum_{ij}{\hat{W}}_{ij}p_{j}\ln\frac{{\hat{W}}_{ij}p_{j}}{{\hat{W}}_{ji}p_{i}}$$

Therefore, failing to recognize that the master equation matrix *W* is the sum of different contributions describing the interaction of the thermodynamic system with various environments, one might underestimate the value of the system entropy production term ${\dot{S}}_{i}$. In Section 5, we show how to compute the different matrices W(k) from our dataset.

## 5. The Case of SARS-CoV-2 Sequence Dataset

In this section, we apply the theory developed before to the case of the SARS-CoV-2 RNA virus, using the sequences dataset downloaded from the National Center for Biotechnology Information (NCBI) public repository. We retrieved the SARS-CoV-2 reference sequence classified as NC045512.2 (the one collected in Wuhan, China, in December 2019), and all the sequences matching the following criteria: same length (29903 base pairs), complete, with no unknown characters, and from a human host. There are about 5600 sequences which constitute the dataset under study in this work.

### 5.1. Computation of Markov Matrix *P* from Data

Let $x=(x_{1},\ldots ,x_{N})$, N=29903, be the reference sequence and let $y=\left(y_{1},\ldots ,y_{N}\right)$ be a mutated sequence. We define for i,j∈E the (empirical) frequency vector associated with *x* and *y*

$$q_{i}=\frac{n_{i}\left(x\right)}{N},\;p_{i}=\frac{n_{i}\left(y\right)}{N}$$

**Figure 4:**
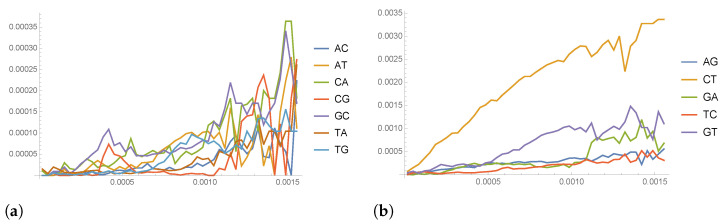
(**a**) Plot of the value of some of the entries of matrix P(k) as a function of the Hamming distance classes k/N; (**b**) the same as in (**a**), showing the entries that gives the major contributions (ten times higher than in (**a**)).

Therefore, the empirical matrix of conditional probabilities can be defined as

$$P_{ij}\left(x,y\right)=\frac{n_{ij}\left(x,y\right)}{n_{i}\left(x\right)}=\frac{n_{ij}\left(x,y\right)}{q_{i}}$$

where $n_{ij}\left(x,y\right)$ is the number of times the base $x_{\alpha }=i$ is mutated in the base $y_{\alpha }=j$ for α=1,…,N. The quantity $d_{H}=0,1,2,\ldots$

$$d_{H}\left(x,y\right)=N-\sum_{i\in E}n_{ii}\left(x,y\right)$$

is the number of errors in the copying of the *x* sequence into *y*, and is called the Hamming distance [16] between the two sequences. Note that the Hamming distance is nonzero for two sequences, which differ by a simple base order exchange, whereas the relative entropy distance is zero in this case, since the base frequencies are unchanged. The Hamming distance thus gives a finer measure of discrepancy between two sequences. We have partitioned our dataset of about 5600 sequences in disjoined classes $D_{H}\left(0\right),D_{H}\left(1\right),\ldots$ of sequences, having the same Hamming distance k=0,1,…, from the reference sequence *x*. We obtained 48 classes, and we define the averaged matrix over class *k* as

(23) $$P{\left(k\right)}_{ij}=\frac{1}{|D_{H}\left(k\right)|}\sum_{y\in D_{H}\left(k\right)}\frac{n_{ij}\left(x,y\right)}{q_{i}}$$

where $|D_{H}\left(k\right)|$ denotes the cardinality of $D_{H}\left(k\right)$. Correspondingly, we define W(k)=P(k)−I. In Figure 4, we have plotted the value of the entries of matrix P(k) as a function of the Hamming distance classes k/N. We see that the major contributions to *P* come from the conditional probabilities C→T (i.e., $P_{CT}$), G→T and G→A, giving another confirmation of the above mentioned C–T bias.

### 5.2. Mean Field Dynamics and Entropy Rate

In this section, we study the entropy evolution by comparing the minimal entropy curve with the mean field dynamics (14) for W=W(k) (see Figure 3a). We see that in the upper part of the curve, the two curves are close to each other, due to the fact that the high sequence length N≈ 30,000 guarantees that the mean field dynamics is a good approximation of the Markovian dynamics. In the lower part of the curve, we see that the mean field solution (blue curve) prescribes a system entropy, which is higher than the minimal entropy curve. The difference is due to the non-negative entropy production term ${\dot{S}}_{i}>0$, while the fact that the entropy is globally decreasing is due to the mutation bias, which can be described as an interaction with a cold environment causing a negative entropy flux ${\dot{S}}_{e}<0$. The better fit of the mean field dynamics is consistent with the fact that the theoretical minimum entropy curve reflects only the knowledge of *q*, while the mean field model of mutation dynamic requires the knowledge of *q* and the Markov matrix *P* of transitions.

**Figure 5:**
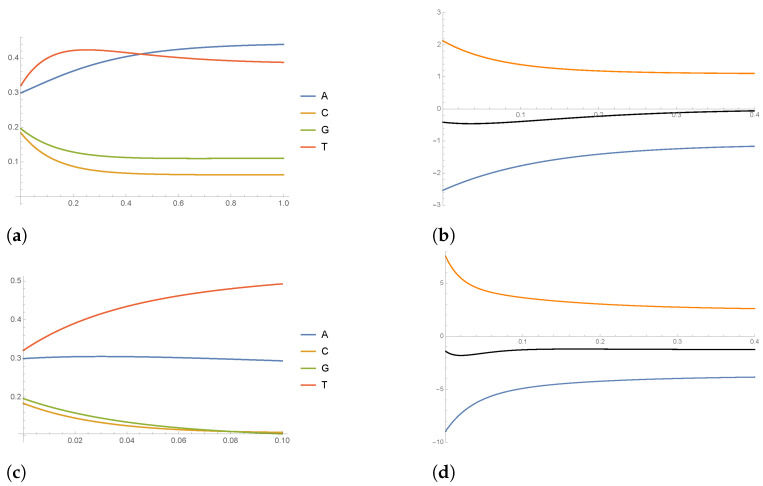
(**a**) Plot of the solution p(t) of the mean field dynamics for W(1); (**b**) plot of entropy rate $\dot{S}$ (black curve), internal entropy rate ${\dot{S}}_{i}$ (orange curve) and entropy flow rate ${\dot{S}}_{e}$ (blue curve) along the solution of the mean field dynamics for W(1); (**c**) the same as in (**a**) for W(40); (**d**) the same as in (**b**) for W(40).

From Figure 3b, we also see that the entropy production term is higher in the case where the dynamics in (14) are described by W(40) with respect to W(1) (compare also Figure 5b,d). This is probably due to the fact that W(40) is averaged over a class of sequences $D_{H}\left(40\right)$, which contains more kind of mutations than $D_{H}\left(1\right)$ (see Section 5.1 above for the definition of $D_{H}$); therefore, it is likely that W(40) “contains” the interaction with multiple environments, a situation that can be described from a theoretical point of view along the lines of Section 4.1. To conclude, in Figure 5, we show the time-evolution of mean field (master equation) dynamics and the time-evolution of the various entropy rate terms $\dot{S}$, ${\dot{S}}_{i}$ and ${\dot{S}}_{e}$.

## 6. Conclusions

In this paper, we have presented an analysis of the mutations in the SARS-CoV-2 RNA sequences. Unlike the majority of genetic studies, which focus on the detailed functional analysis of very specific regions of the sequences, we have considered only the sequence base frequency as relevant information. Using a literary analogy, we discarded the poetry in the book, and we concentrated only on the differences due to typographic errors between the millions of printed copies. We can thus understand some features of the printing machine, and discover that is biased towards some kind of errors. The functioning of the printer can be described quantitatively by a probabilistic model, which is amenable to a stochastic thermodynamic interpretation. We modeled the accumulation of mutations in the RNA sequence as the slow drift of the probability p=p(t) describing a four-state thermodynamical system in contact with a thermal bath from the initial q=p(0). The evolution of the probability can be described as the mean field evolution of a Markov chain, whose matrix *P* is derived from data, and it describes the existence of a mutation bias since the entropy is decreasing. It is remarkable that, for SARS-CoV-2, the entropy decrease closely follows a theoretically computable lower bound. As far as we know, this is result is new. We think that this simple model can complement classical approaches to the problem of describing genetic variability.

Indeed, our approach is not confined to the study of genetic sequences, and it is virtually applicable to any dynamical system described by a vector field $\dot{x}=X\left(x,t\right)$ over a manifold *M* and a finite partition *E* of *M* (coarse graining). The coarse-grained system evolution is described by a sequence $x=(x_{1},\ldots ,x_{N})$, $x_{i}\in E$, and the probability vector *q* is the so-called occupation measure of *x*. If we add a noise or drift term to the deterministic evolution *X*, then we have a set of perturbed trajectories $y=(y_{1},\ldots y_{N})$ fluctuating around *x*. One could retrieve some aspects of the evolution of the perturbed system from a record of collected trajectories along the lines described in this work.

## Data Availability

The data that support the findings of this study are openly available at NCBI (National Center for Biotechnology Information) database (https://www.ncbi.nlm.nih.gov).

## Appendix A

### Mean Field Approximation of Markovian Dynamics

We adapt the argument contained in [13] to our needs. Let us consider a population of *N* identical particles subdivided between *k* urns. The probability distribution $p=\left(p_{1},\ldots ,p_{k}\right)=\left(\frac{n_{1}}{N},\ldots ,\frac{n_{k}}{N}\right)$ is called the occupation measure. We have $p\in S_{Q}^{k}$, where $S_{Q}^{k}$ is the *k*-dimensional simplex with rational values, which is a dense subset of $S_{R}^{k}$. Let us fix a time step Δt and consider the sequence of random variables $X_{n}=p\left(t+n\Delta t\right)\in S_{Q}^{k}$. Let us suppose that at every time step, only one particle moves from from urn *i* to urn *j*, so that

$$p\left(t+\Delta t\right)=p\left(t\right)+\frac{1}{N}\left(e_{j}-e_{i}\right)\;\forall i,j=1,\ldots ,k$$

where $e_{i}$, i=1,…k are the unit vectors of the canonical base in $R^{k}$. Set Δt=1/N, and define the conditional probability

$$P_{ij}\left(p\right)=\mathrm{Prob}\left(p\left(t+\frac{1}{N}t\right)=p+\frac{1}{N}\left(e_{j}-e_{i}\right)|p\left(t\right)=p\right)$$

Let us suppose that they are continuous functions of *p* and independent of *t* and *N*. In this way, the sequence of random variables defines a discrete time Markov chain. Let us define the function on $S_{Q}^{k}$

$$F_{i}\left(p\right)=in\left(i\right)-out\left(i\right)=\sum_{j=1}^{k}P_{ij}\left(p\right)-P_{ji}\left(p\right),\;i=1,\ldots ,k$$

Since $\sum_{i}F_{i}\left(p\right)=0$, $F\left(p\right)\in T_{p}S_{Q}^{k}$, the tangent space to $S_{Q}^{k}$. Since F(p) is continuous and it is defined on a dense subset of $S_{R}^{k}$ it can be extended to $S_{R}^{k}$, which is compact. Therefore, F(p) is a Lipschitz continuous vector field, and one can consider the O.D.E. and the Cauchy problem

$$\dot{p}=F\left(p\right),\;p\left(0\right)=q$$

The vector field *F* is called the mean field dynamics (or the fluid limit approximation) associated with the discrete Markov chain. Let p(t,q) be the solution, and denote with $p_{c}\left(t\right)$ the stochastic process with continuous time interpolating the Markov chain. Moreover, let us consider the random variable

$$D_{T}\left(q\right)=max\{|p_{c}\left(t\right)-p\left(t,q\right)\left|,t\in 0,T\right\}$$

The following large deviation type estimate is given in [13].

**Proposition** **A1.**
*There exists a C>0 such that for all ε>0, T>0 and sufficiently large N, one has*

$$Prob\left(D_{T}\left(q\right)\geq \varepsilon \;|\;p\left(0\right)=q\right)\leq 2ke^{-C\varepsilon^{2}N}$$

For the Markov model presented in Section 3 (see (13)), we have

$$F_{i}\left(p\right)=\sum_{j}p_{j}P_{ji}-p_{i}P_{ij}={\left(\left(P^{T}-I\right)p\right)}_{i}.$$
